# Transcriptome characterization and expression profile of *Coix lacryma-jobi* L. in response to drought

**DOI:** 10.1371/journal.pone.0256875

**Published:** 2021-09-03

**Authors:** Guidong Miao, Yan Qin, Jihua Guo, Qingxia Zhang, Yingying Bao

**Affiliations:** School of Biology and Chemistry, Xingyi Normal University for Nationalities, Xingyi, Guizhou Province, China; Nazarbayev University, KAZAKHSTAN

## Abstract

*Coix lacryma-jobi* L. is a very important economic crop widely cultivated in Southeast Asia. Drought affects more than four million square kilometers every year, and is a significant factor limiting agricultural productivity. However, relatively little is known about how *Coix lacryma-jobi* L. responds to drought treatments. To obtain a detailed and comprehensive understanding of the mechanisms regulating the transcriptional responses of *Coix lacryma-jobi* L. to drought treatment, we employed high throughput short-read sequencing of cDNA prepared from polyadenylated RNA to explore global gene expression after a seven-day drought treatment. We generated a de novo assembled transcriptome comprising 65,480 unique sequences. Differential expression analysis based on RSEM-estimated transcript abundances identified 5,315 differentially expressed genes (DEGs) when comparing samples from plants following drought-treatment and from the appropriate controls. Among these, the transcripts for 3,460 genes were increased in abundance, whereas 1,855 were decreased. Real-time quantitative PCR for 5 transcripts confirmed the changes identified by RNA-Seq. The results provide a transcriptional overview of the changes in *Coix lacryma-jobi* L. in response to drought, and will be very useful for studying the function of associated genes and selection of molecular marker of *Coix lacryma-jobi* L in the future.

## Introduction

*Coix lacryma-jobi* L. (*Coix*) (2n = 20), also commonly known as Job’s Tears, belongs to the family Poaceae and is the closest group to the genera *Zea*, *Tripsacum*, and *Sorghum* [1]. It is widely cultivated as a food and medicinal plant in East and Southeast Asian countries [2]. *Coix* has higher protein content and oil content than other cereals, which makes it a good source of nutrition for humans and animals [3]. Because of these valuable traits, *Coix* is cultivated as a minor crop in Asia. The current planting area of *Coix lacryma-jobi* L. in China is estimated to be around 73,000 ha, providing a grain yield of 0.22 million tons [4]. Guizhou province (GZ), and particularly southwest Guizhou, has become the largest producer and distribution center for *Coix lacryma-jobi* L. in Southeast Asia [4, 5].

Many studies have appeared concerning the genetic diversity of *Coix lacryma-jobi* L. The first report Li et al. employed RAPD (random amplified polymorphic DNA) analysis to provide a genetic evaluation of *Coix* germplasm [6]. Ma et al. (2010) extended this to 79 *Coix lacryma-jobi* accessions, finding that accessions from Guangxi Province in China possessed greater genetic diversity than accessions originating from Korea [7]. Guo et al. evaluated 22 *Coix lacryma-jobi* accessions using SSR (simple sequence repeats) developed from maize and rice [8]. Wang et al. (2015) studied the genetic diversity of 25 *Coix lacryma-jobi* using SRAP (sequence-related amplified polymorphism) markers, and found the 25 populations of *C*. *lacryma-jobi* could be clustered into four groups [9]. Fu et al. (2019) assessed the genetic diversity and population structure of 139 genotypes of *Coix lacryma-jobi* from China using AFLP (amplified fragment length polymorphism) markers, and found the overall genetic diversity of 139 *Coix lacryma-jobi* L. was relatively low, although *Coix lacryma-jobi* from GZ showed a higher level of genetic diversity compared to genotypes from other three regions [10]. Kang (2018) identified tissue-specific genes from the adlay leaf, root, and young and mature seed using long-read isoform sequencing (Iso-Seq) and short-read RNA-Sequencing (RNA-Seq) [11].

Plant environmental stresses include drought, salt and cold, which seriously threaten crop yield. Most cereals, such as wheat and rice are sensitive to drought stress. Plants have evolved appropriate strategies to cope with water stress in order to ensure their survival and reproduction. Plant have evolved many mechanisms to respond to drought, such as sensing water turgor, binding surrounded water, and changing cell membrane structure and permeability [12]. High-throughput transcriptome sequencing has been widely used to study gene expression patterns and simultaneously identify mutations, sequence variations, and alternative splicing variants [13]. Moreover, it also allows us to profile DEGs under different physiological conditions, such as drought [14, 15].

Although good progress has been made concerning uncovering genetic diversity in *Coix*, how this species responds to the stress has not been studied in any detail at the level of the transcriptome. The present study, which obtained more than 40 million clean reads, is the first high-throughput sequencing analysis of the transcriptome of *Coix lacryma-jobi* to annotate drought-associated genes expressed in the plant. Our aim was to identify novel genes that can be used to better understand the basic biological mechanisms of response to drought for this species. A comprehensive understanding of drought mechanisms and the regulatory pathways in *Coix* could gain the desired traits in *Coix* leading to drought resistance. In addition, this will provide data resources for the drought investigations of gene expression and gene function in the future.

## Materials and methods

### Plant growth and treatments

Seeds of *Coix lacryma-jobi* derived from cultivated species were obtained from Qinglong, in the southwestern region of Guizhou province, China. The field-collected ten seeds from different mothers were surface-sterilized with a 75% ethanol solution, rinsed with sterilized distilled water, and sown in sterilized soil for germination. The seedlings were grown in plastic pots (800 mL) covered with commercially bought peatsoil and loess (3:2), under a 16/8 h photoperiod at 25°C (day) and 18°C (night). For the control group, plants were taken at the 3-leaf stage, and were watered with 300 ml of water, maintaining 80%-85% soil humidity. For the treatment group, plants were also taken at the 3-leaf stage, but the plants were grown without watering for seven days until the leaves were wilted and obviously curled.

### Sample collection and RNA preparation

Whole leaves of the control and treatment groups, each with five plants were collected and were separately frozen in liquid nitrogen and stored at -80°C prior to RNA extraction. Total RNA was isolated using TRIzol reagent (Invitrogen^TM^, Carlsbad, CA, USA) following the manufacturer’s instructions. A NanoDrop 2000 (Thermo Scientific, Waltham, MA) and Bioanalyzer 2100 (Agilent Technologies, Waldbronn, Germany) were used to determine total RNA concentrations and to assess RNA integrity, respectively. For each sample, at least 20μg of total RNA was sent to Vazyme BioTechnologies (Nanjing, Jiangsu, China) for Illumina sequencing.

### Sequencing and assembly

The total mRNAs isolated from each group were pooled together as one combined sample. Thus, two combined samples were obtained, denoted as Drought-Treated and Control accordingly. Poly (A) mRNA was isolated using oligo-dT beads (Qiagen, Dusseldorf, Germany). Fragmentation buffer was used to convert all mRNA into short fragments. Random hexamer-primed reverse transcription was employed for the first-strand cDNA synthesis. RNase H and DNA polymerase I were used for the production of double-stranded cDNAs. The QIAquick PCR extraction kit (Qiagen, Inc., Dusseldorf, Germany) was employed for cDNA purification. The purified cDNAs were washed with EB buffer for end repair, poly (A) addition, and ligation to sequencing adapters. Agarose gel electrophoresis was then used to isolate fragments suitable in size for sequencing, which were extracted from the gels, and amplified by PCR for the final cDNA library preparation. The library was sequenced on the Illumina HiSeq 2500 with paired-end technology by Vazyme BioTechnologies (VAHTS^TM^ mRNA-seq v2 Library Prep Kit). The Illumina GA processing pipeline was used for image analysis and base calling. All the raw RNA-Seq data can be found in the National Genomics Data Center BioProject database with the accession number PRJCA 004388.

Before further analysis, the raw reads were filtered to remove adaptor sequences, low-quality reads, and reads containing poly-N, using CutAdapt (http://code.google.com/p/cutadapt/) and Btrim [16]. Then, all clean reads of the two libraries were pooled together and assembled into contigs with Trinity software (version 2.1.1) [17]. Contigs longer than 200 bases were considered for the following analysis after assembly. Unigenes were formed when contigs could not be further extended on either end. These unigenes were further spliced by a k-mer cut-off value of 25 and then assembled to acquire maximum length non-redundant unigenes, using the TGICL clustering software (J. Craig Venter Institute, Rockville, MD).

### Functional annotation of unigenes

Blastx analysis was run with an E-value 10^−5^ between the unigenes and the databases of Non-Redundant proteins (NR), Swiss-Prot, the Kyoto Encyclopedia of Genes and Genomes (KEGG), Clusters of Orthologous Groups (COG), and Gene Ontology (GO) [18]. Blastn was performed to align these unigenes to the nucleotide (NT) database, searching proteins with the highest sequence similarity to the given unigenes with their protein functional annotations. GO annotation of these unigenes was run with WEGO [19], based on the results of the National Center for Biotechnology Information NR database annotation.

### Identification of differentially expressed genes

To identify differentially expressed genes (DEGs) in response to water stress, the expression levels of unigenes were calculated in FPKM (fragments per kilobase of exon per million fragments mapped reads) values [20] using RSEM software [21], and the expression changes were determined by comparing the drought samples with the control samples. Those unigenes displaying fold-changes of more than two, and a false discovery rate (FDR) less than 0.001, were regarded as being significantly DEGs using edgeR [22].

Gene Ontology analyses were performed to identify the enrichment of DEGs in GO terms. The DEGs in the GO analysis were enriched using WEGO [19]. The GO enrichment analysis for the DEGs were performed by conducting hypergeometric tests with the entire *Coix* leaf transcriptome set as the background. A corrected p-value (q-value) ≤ 0.05 was chosen as the threshold for significantly enriched GO terms.

COG annotation of the DEGs was performed using the Blast software, and GO enrichment analysis (P value 0.05) using GOseq with the Wallenius noncentral hypergeometric distribution model.

### Identification of simple sequence repeats and single nucleotide polymorphisms

MicroSAtellite (MISA) software (https://webblast.ipk-gatersleben.de/misa/) was used to identify simple sequence repeats (SSRs) markers in all unigenes of pooled samples. The search criteria were as follows: dinucleotide repeats 6, trinucleotide to hexanucleotide repeats 5 and the largest interval between two SSRs 100 bases.

All unigenes of each group were employed as reference sequences to detect potential single nucleotide polymorphisms (SNPs) using SOAPsnp (http://soap.genomics.org.cn/soapsnp.html) [23]. Based on the different bases found at any one position in the assembled sequences from the same unigenes, SNPs sites were predicted.

### Validation of RNA-Seq by qPCR

We selected 5 genes, each with four biological replicates, for the validation of RNA-Seq by qRT-PCR. Total RNA samples (0.5ug) were reversed-transcribed using oligo (dT) and Superscript II reverse transcriptase (Thermo Fisher Scientific) in a total volume of 10uL. The program was as follows: 42°C for 60 min and 70°C for 15min. The cDNA samples were then diluted to 25uL and kept at -20°C. qRT-PCR was performed using a CFX Connect real-time system (Bio-Rad) using the Talent qPCR Premix (SYBR Green) kit (TIANGEN Biotech, Beijing, China) following the manufacturer’s instructions. The specific primers were designed online (https://www.ncbi.nlm.nih.gov/tools/primer-blast/) according to the following parameters: primer melting temperature of 57–63°C with the optimized 60°C, the PCR product size of 90-180bps. The specific primer sequences of the selected genes for qRT-PCR validation were designed based on the divergent regions among the orthologous genes and listed in S1 Table. Melting curves were assessed and only primers with single peak were selected. Three technical replicates were used for the samples with 18S rRNA as the reference gene and the quantification of qPCR results for each unigene were calculated using the delta-delta Ct (2^-ΔΔCt^) method. For each gene, a linear standard curve was constructed using a serial dilution of a specific cDNA standard. The transcript levels of all unknown samples were determined according to the linear standard curve. PCR amplication efficiency (E) was calculated as follows: E = (10^[-1/slope]^-1)×100.

Each PCR reaction volumes were set at 20uL. The mix contained 10uL of 2×Talent qPCR PreMix, 0.6uL of forward and reverse primers (10uM), 8.7uL of RNase-free ddH_2_O and 0.7uL of cDNA template. PCR cycling was performed according to the following programs: 3min at 95°C followed by 40 cycles of 5s at 95°C and 15s at 60°C. Melting curve cycling consisted of: 65°C for 5s and then 0.5°C increment for 5s until 95°C.

## Results and discussion

### Transcriptome sequencing and assembly

**Fig 1:**
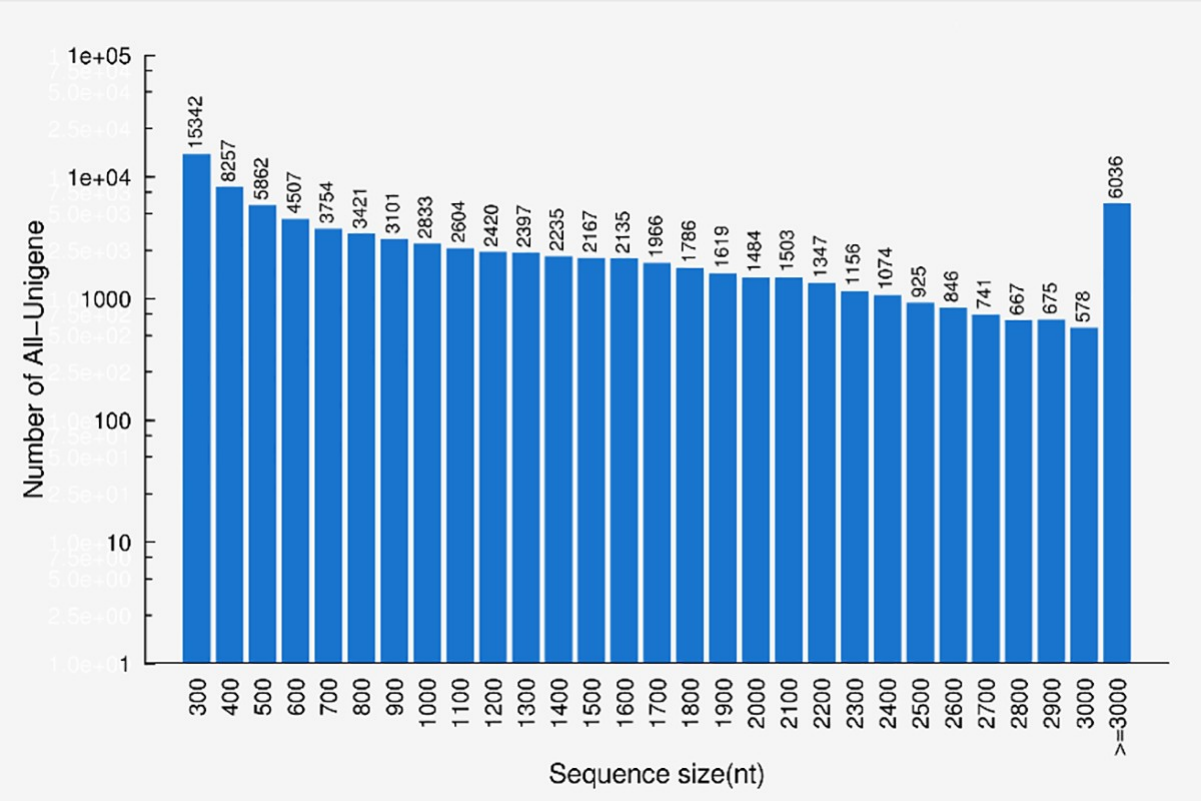
Length distribution of the assembled contigs and unigenes.

Total RNA from *Coix* leaves collected from 10 individual plants was sequenced in several Illumina HiSeq 2500 runs. A total of 44.9 million and 42 million raw reads were generated from drought treatment and control groups, respectively (Table 1). After removing the adaptor sequences and filtering out low-quality reads, we obtained 43.5 million and 40.8 million clean reads from the drought treatment and the control samples, respectively. A total of 127,704 contigs (average length = 423nt) from pooled samples were generated and the N50 sizes were 899nt. Finally, we obtained 83,438 unigenes and the N50 size and the mean size were 1,918 and 1,195nt, respectively. Fig 1 shows the length distribution of all the contigs and unigenes, ranging from 200 nt to more than 3,000 nt.

**Table 1:** Summary of RNA sequencing of the *Coix lacryma-jobi* L.

	Drought treatment	Control	All
Total raw reads	44,879,148	42,031,694	
Total clean reads	43,473,808	40,775,620	
	*Contig*		
Total number			127,704
Total length, nt			54,055,581
Mean length, nt			423
N50			899
	*Unigene*		
Total number			83,438
Total length, nt			99,721,439
Mean length, nt			1,195
N50			1,918
Total consensus sequences			83,438
Distinct clusters			36,138
Distinct singletons			47,300
	*Annotation*		
NR			56,202
NT			61,417
Swiss-Prot			36,846
KEGG			36,515
COG			24,360
GO			42,174
All annotated unigenes			65,480

Similarity analysis of all assembled unigenes was performed by BLAST. A total of 65,480 unigene sequences were hit to the protein databases along with their protein functional annotations, which included NR, KEGG, Swiss-Prot, COG, and GO (E-value<10−^5^) using blastx, and NT (E-value<10^−5^) using blastn, accounted for 78.5% of all the assembled unigenes. A total of 56,202, 61,417, 36,846, 36,515, 24,360, and 42,174 unigenes were annotated to the NR, NT, Swiss-Prot, KEGG, COG, and GO databases, respectively (Table 1). A total of 56,101 coding sequences (CDS) were mapped to the protein database and 1,640 CDS were predicted in the protein coding region prediction analysis.

### Functional annotation and classification of unigenes

**Fig 2:**
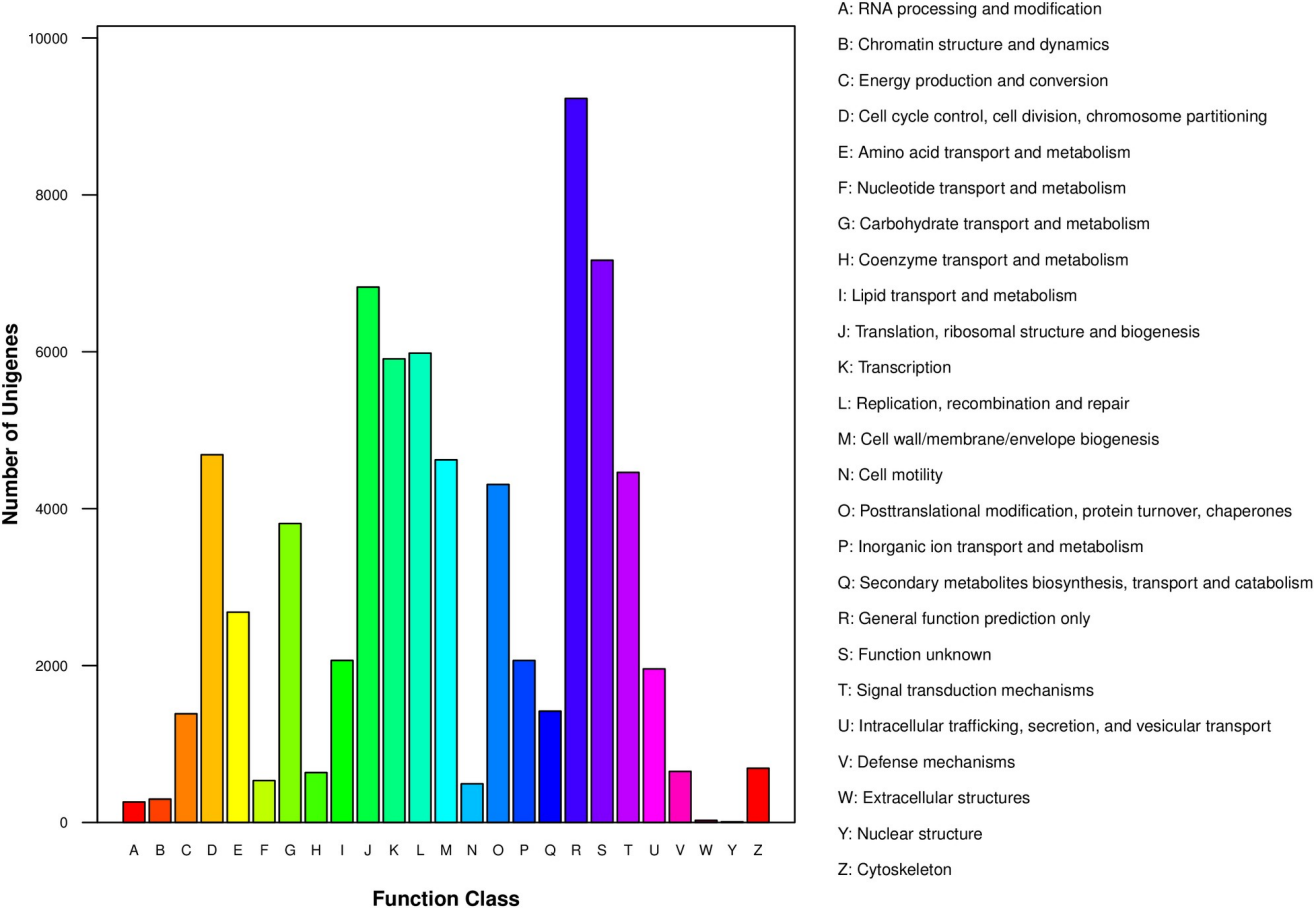
The Clusters of Orthologous Groups (COG) functional classification of all unigene of *Coix lacryma-jobi* L.

The unigenes of *Coix lacryma-jobi* were then subjected to functional annotation and classification analysis. All results were summarized in S2 Table. In the COG classification system, totally 24,360 (29.20%) unigenes were annotated and classified into 25 COG groups (S3 Table). The largest cluster was “the general function prediction only (R)” and the second was “function unknown (S)”, which indicated that most genes’ functions were predicted by bioinformatics and needed to be confirmed by experiment. The following clusters were “translation, ribosomal structure and biogenesis (J)”, “replication, recombination and repair (L),” and “transcription (K)” (Fig 2).

**Fig 3:**
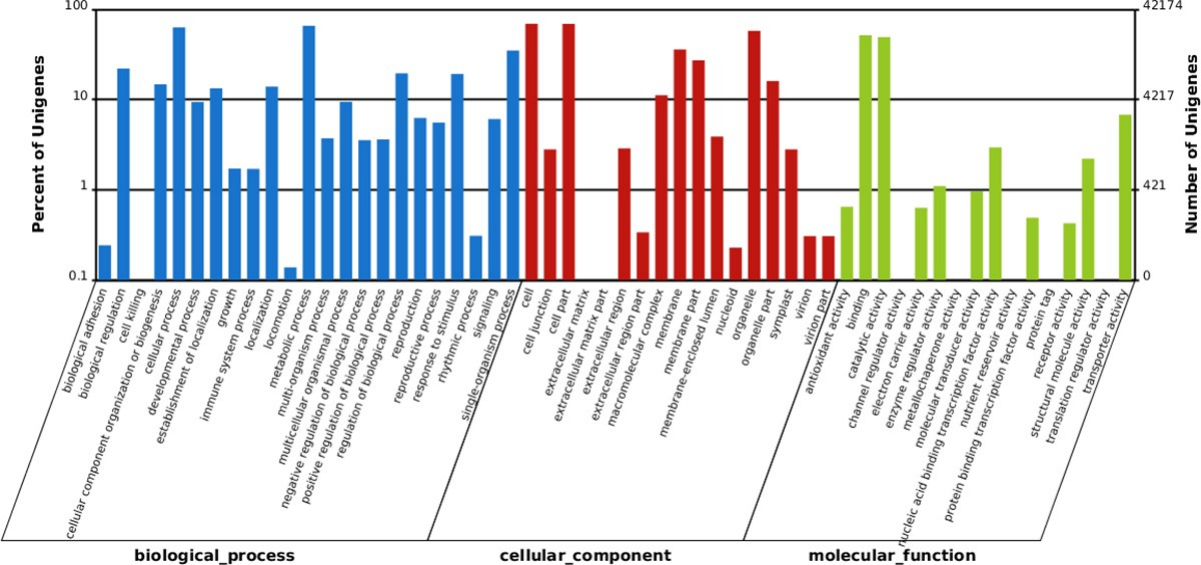
Analysis of Gene Ontology (GO) functional enrichment of assembled unigenes of *Coix lacryma-jobi* L. The functions of genes identified covering three main categories, biological processes (BP), cellular components (CC), and molecular functions (MF), are indicated.

GO enrichment analyses were performed to classify the putative functions of unigenes. Based on sequence homologies, the unigenes were separated into three main categories (biological processes, cellular components and molecular functions), which annotated to 56 functional groups of GO classification (Fig 3). Within biological processes, a high percentage of transcripts fell into the following functional groups: cellular process (26,615 unigenes), metabolic process (27,739 unigenes) and single-organism process (14,741 unigenes). In the cellular component category, cell (29,207 unigenes), organelle (24,364 unigenes) and cell part (29,137 unigenes) were enriched, whereas within the molecular function category binding (21,793 unigenes), catalytic activity (20,774 unigenes), and transporter activity (2,859 unigenes) were enriched (S4 Table). The GO functional enrichment analysis indicates that the changes in biological process may be very important in drought responses.

COG and GO analyses were performed to describe the gene products. In our study, a total of 24,360 and 42,174 unigenes were annotated in COG and GO databases, respectively. The categories “the general functional prediction only”, “function unknown”, and “translation, ribosomal structure and biogenesis”, were enriched in COG analysis. In GO assignments, “metabolic process”, “catalytic activity” and “transporter activity” were significantly enriched. Genes in these functional groups were strikingly more abundant, which provides basic information for further analysis of drought stress mechanisms in *Coix lacryma-jobi*. The Kyoto Encyclopedia of Genes and Genomes (KEGG) analysis was performed to identify their function and metabolic pathway. The metabolic pathway, such as RNA transport, mRNA surveillance pathway, biosynthesis of secondary metabolites and glycerophospholipid metabolism were the top five KEGG metabolic pathway. Some of the unigenes were mapped to several pathways related to stress, such as plant hormone signal transduction, plant–pathogen interaction and ubiquitin-mediated proteolysis. All the unigenes that mapped to the KEGG pathways were presented in S5 Table.

A large number of key genes and numerous important pathways that may be correlated with drought were obtained in the current transcriptomic data. KEGG analyses indicate secondary metabolites biosynthesis such as benzoxazinoids synthesis had been the major pathway during the drought treatment.

**Fig 4:**
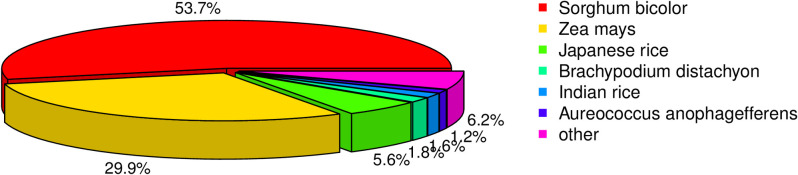
The species distribution based on the BLAST of NR database.

A total of 56,202 unigenes matched sequences from 542 species in the NR database (S6 Table). Most matches were corresponding to *Sorghum bicolor* (53.7%), *Zea mays* (29.9%), *Japanese rice* (5.6%), *Brachypodium distachyon* (1.8%) and *Indian rice* (1.6%) (Fig 4).

### Functional annotation and analysis of DEGs

Using edgeR software, we obtained a total of 5,315 differentially–expressed transcripts which were successfully annotated in the NR database (S7 Table). Of all the unigenes, 3,460 increased in abundance while 1,855 decreased. Some of the DEGs were water stress-related genes, such as *dehydration-responsive element binding protein* and *dehydrin DHN1* [24], which were up-regulated. Compared with the previous study of Huang et al., in which 1,128 DEGs were identified, the current study detected more DEGs (5,315).

Among all the DEGs, many genes involved in stress-response were up-regulated, such as *cytochrome 450*, which possibly participated in cell death; *heat shock protein 70*; *disease-resistance protein RPM1* and *peroxidase 12*, which is the physiological index of tissue aging. Also, many genes were down-regulated, such as photosynthesis–related genes *light harvesting chrolophyll a/b*; transcription factors *R2R3-MYB WRKY62* and *far-red impaired response protein* (*FAR1*); plant hormone related gene *auxin efflux carrier* and *indole-3-glycerol phosphate lyase*; *UDPG-flavonoid 3-o-glucosyl transferase*, which catalyze the biosynthesis of anthocyanin, *cell-wall invertase* and *senescence-associated protein 15*. These findings suggested that the basic metabolic activity of *Coix* decreased when they were exposed to water stress condition. Another drought-responsive gene, *GAPC1*, has been evaluated for its potential role in drought stress in wheat and *Arabidopsis*, their results indicated that the expression level of *GAPC1* was induced by abiotic stress, and GAPC1 could promote H_2_O_2_ detoxification to enhance drought tolerance [25, 26]. In the present study, the gene *GAPC1* was found to be up-regulated, which is consistent with the studies in Arabidopsis.

**Fig 5:**
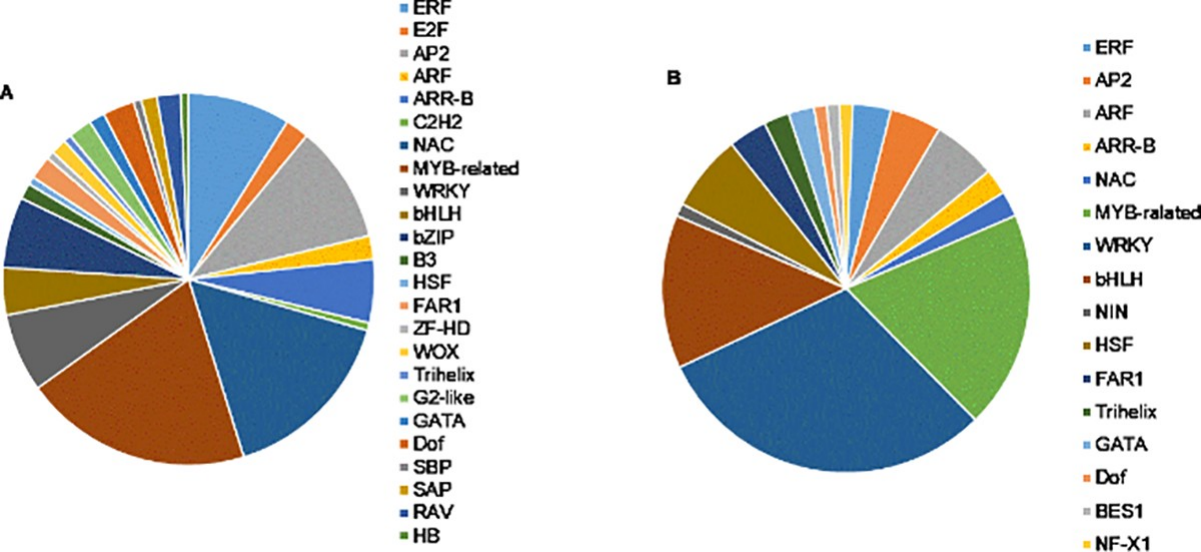
The up- (A) and down-regulated (B) differentially expressed TF families in *Coix lacryma-jobi* L.

Transcription factors (TFs) were one of the most important families regulating the gene expression. In the current study, we obtained 235 TFs of all identified DEGs, with approximately 4.42%, belonging to 40 families based on the PlantTFDB 5.0 [27]. The DE TFs shows both up- and down-regulation patterns, of which the up-regulated TF families including MYB-related (12.3%), NAC (9.8%), AP2 (6.4%) and ERF (5.5%) (Fig 5A) and the down-regulated TF families including WRKY (11.5%), bHLH (5.1%), HSF (2.6%) and ARF (2.1%) (Fig 5B). Many of these TFs have been reported to be responsive to drought in other plants [28–31]. For example, bZIP TFs regulate ABA-related gene expression and AP2 TF family involve in hormonal and abiotic stress responses [32]. Our results identified 16 putative AP2 TFs and 9 putative bZIP TFs, which were up-regulated 12.21-fold and 11.95-fold, respectively.

**Fig 6:**
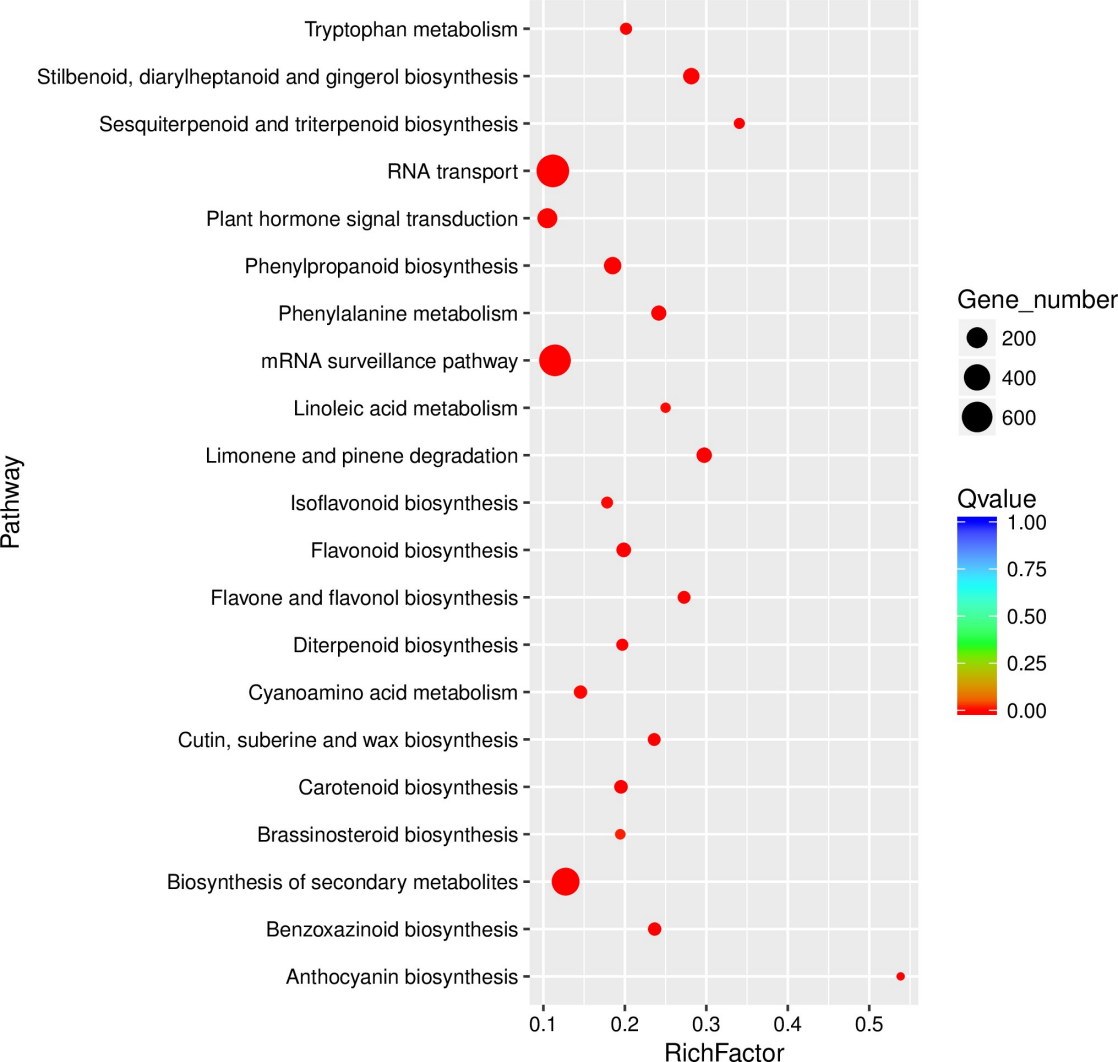
GO term enrichment analysis of DEGs of *Coix lacryma-jobi* L.

DEGs were then further performed GO and KEGG annotations to predict their potential functions and associated metabolic pathways. The GO classifications of the DEGs were presented in Fig 6. In categories of biological processes, cellular process (1,765), single-organism process (1,455), and metabolic process (1,977) were the top three abundant GO function terms, whereas cell (1,933), cell part (1,930), and organelle (1,598) were the top three abundant terms in the categories of cellular component. In categories of molecular function, binding (1,494), catalytic activity (1,561), and transporter activity (242) had the most abundant GO function terms (S8 Table). The GO functional enrichment analysis indicates that the changes in the biological process may be very important in drought responses.

**Fig 7:**
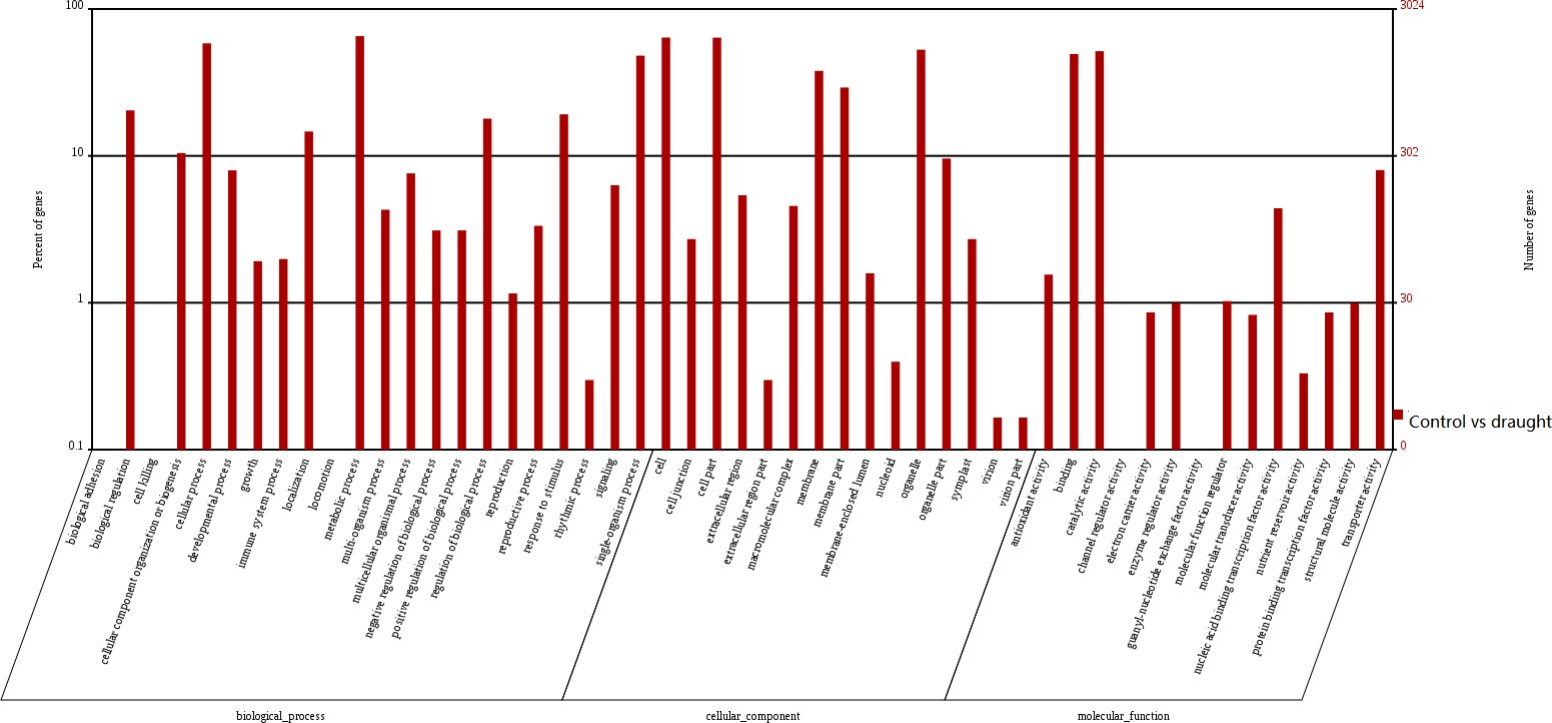
Description of the mapped KEGG pathways of the DEGs in *Coix lacryma-jobi* L. Left: The KEGG pathways; Right: the number of DEGs mapped into each KEGG pathway.

To further investigate the functions of differentially expressed transcripts of Coix in response to drought, we performed enrichment analyses by mapping the sequences to the KEGG database categories. The DEGs with KEGG annotation were assigned to 31 classes with a threshold of P-value<0.05 (S9 Table), mainly related to biosynthesis of secondary metabolites (467), RNA transport (722), mRNA surveillance pathway (652) and plant hormone signal transduction (171). KEGG enrichment analyses also indicated that the DEGs were significantly enriched in the main pathways of metabolism (ko01110), genetic information processing (ko03013) and in the environmental information processing (ko04075), which were closely related to stress (Fig 7).

### Identification of molecular markers

**Fig 8:**
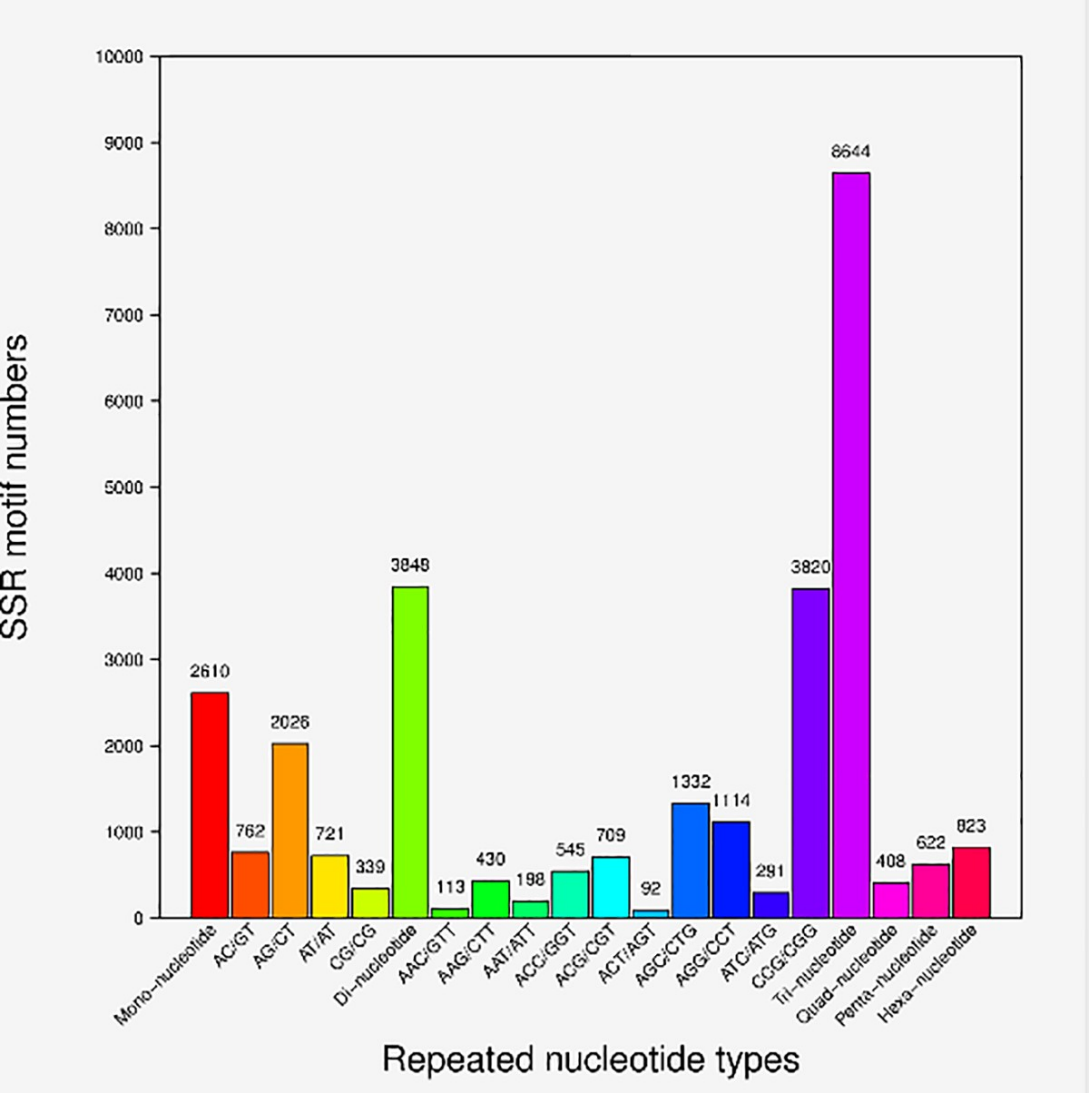
Statistics of Simple Sequence Repeats (SSRs) taxonomy of *Coix lacryma-jobi* L.

The unigenes were used to predict SSRs using the MISA software. A total of 16,955 SSRs were identified from the transcriptome. Trinucleotide repeats (8,644) was the largest fraction of SSRs with CCG/CGG and AGC/CTG motifs, followed by dinucleotide repeats (3,848) and mononucleotide repeats (2610) (Fig 8).

**Fig 9:**
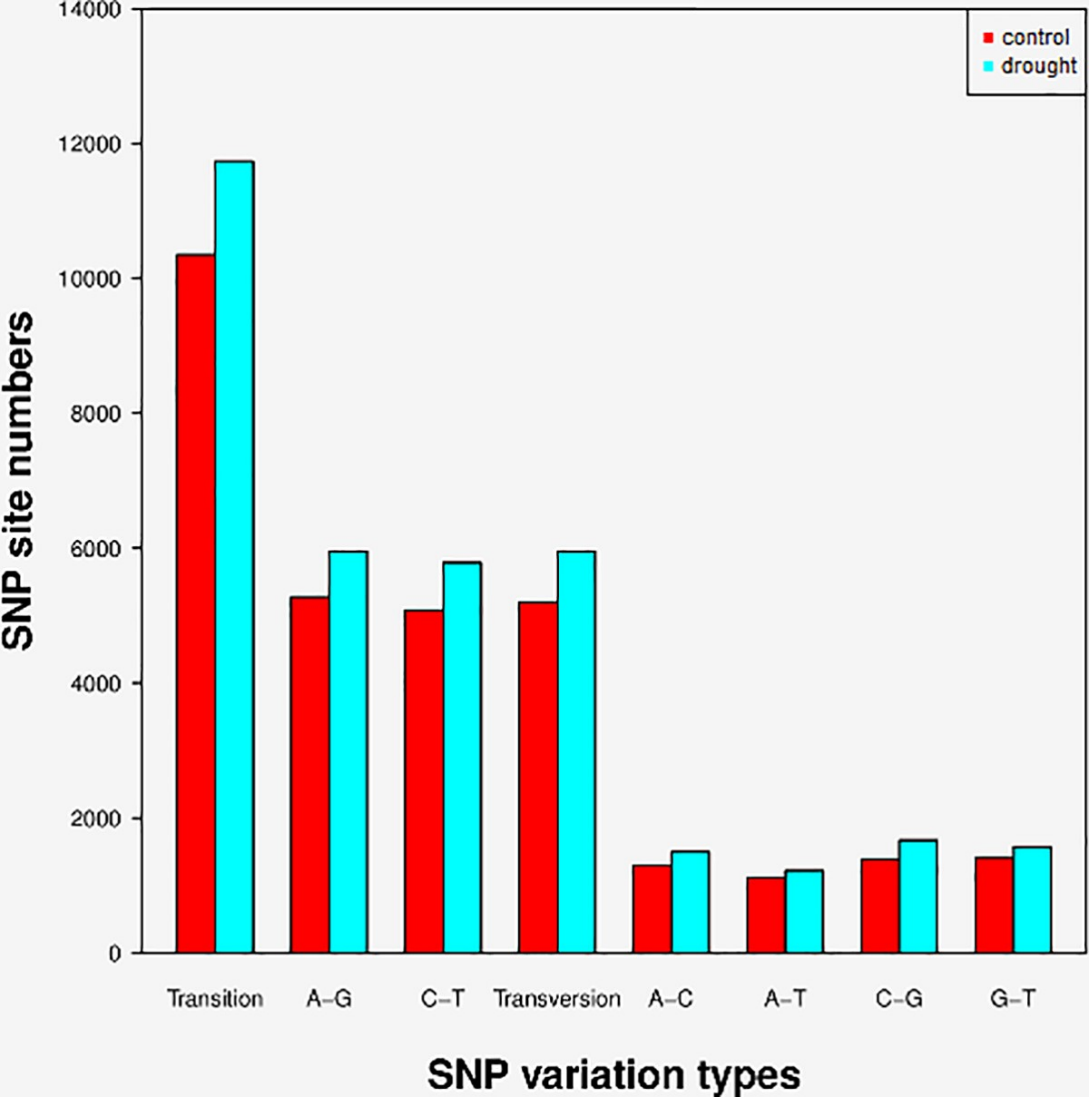
Quantitative statistics of Single Nucleotide Polymorphism (SNP) classification of *Coix lacryma-jobi* L.

A total of 17,690 and 15,549 high quality SNPs were identified from the *Coix lacryma-jobi* L. unigenes derived from the drought treatment and control samples, respectively. The predicted SNP included 11,736 transition and 5,954 transversions in drought sample, while 10,349 transition and 5,200 transversions in control sample. Types A-G and C-T of SNP were the most abundant transition, and the proportions of drought sample was higher than in the control sample. On the other hand, transversion SNP types, A-C, A-T, C-G and G-T, the level of abundance were all similar between the two groups (Fig 9).

SNPs are widely used in whole-genome studies and trait-mapping due to their extensive distribution and abundance [33], so they are potential markers in organisms lacking fully-annotated genomes [34–36]. Many molecular markers have been found now, among which SSRs are widely used in parentage analysis, quantitative trait loci (QTL) association and population genetics, by virtue of their codominant and polymorphic nature [37, 38]. Meanwhile, SSRs and SNPs are important molecular markers in studies of population genetic analysis and breeding. In the present study, 16,955 SSRs were predicted in the transcriptome data, and a total of 17,690 and 15,549 SNPs from the sequencing data of drought treatment and control samples, respectively. The SSRs and SNPs identified in this study will be valuable resources to study population genetics, QTL correlation, genetic mapping, and novel gene exploration in *Coix*.

### Validation of RNA-Seq results by qPCR

**Fig 10:**
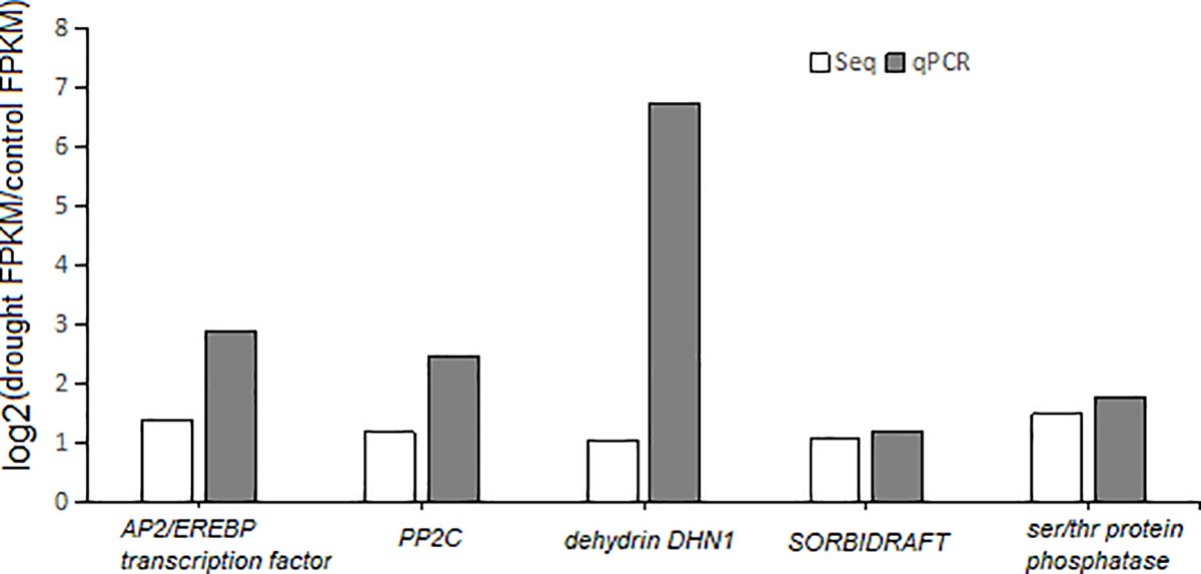
Expression levels of candidate genes in qRT-PCR and RNA-Seq.

The RT-qPCR followed the guidelines of the MIQE (Minimum Information for Publication of Quantitative Real-Time PCR Experiments) [39]. qPCR was performed in selected five genes, including *drought-related AP2/EREBP transcription factor*, *dehydrin DHNI*, *protein phosphatase 2C*, *protein SORBIDRAFT and serine/threoine protein phosphatase*, to validate the RNA-Seq results. The expression patterns of the qPCR were consistent with the bioinformatics data (Fig 10), which validated the results of the RNA-Seq.

In the qPCR, we selected five drought-related genes to validate the results of RNA-Seq. The *Dehydrin DHN1* gene, which is responsive to abscisic acid (ABA), plays a vital role in drought stress conditions in *Glycine max* and *Fagus sylvatic*a seeds [40, 41]. The functions of the Dehydrin DHN1 protein have been reported in maize [42]. In the present study, this gene was found to be significantly up-regulated, which indicated that the plant hormone ABA might be involved in the regulation of the *Coix lacryma-jobi* drought response.

The family of APETALA2/ ethylene responsive element binding proteins (AP2/EREBP)-like proteins are the prototypic members of a family of transcription factors unique to plants, they play a variety of roles throughout the plant life cycle, especially to respond to various types of biotic and environmental stress. The molecular characteristics and diverse functions have been reported in Arabidopsis [43] [44]. Other interesting study reported that this gene is involved in mediating cuticular permeability, sensitivity to abscisic acid, and drought resistance by regulating wax biosynthesis [45]. In our study, this transcription factor was also highly expressed in the qPCR data. This indicates that AP2/EREBP is a vital transcription factors of *Coix* that is involved in the signaling pathway to activate the drought-responsive genes to enhance the drought stress tolerance.

Our analysis has identified hundreds of genes encoding transcription factors that are either induced or repressed by drought stresses [46]. The genes activated by drought include those involved in mechanisms designed to avoid water loss, protect the cellular machinery, and repair damage [47–49]. One response to plant water deficit is the synthesis of osmolytes [50]. Transport proteins, ion channels and carriers also play an important role in water deficit avoidance or osmoregulation [51].

According to the study of Huang (2017), 1053 annotated genes related to drought in *Coix* transcriptome were reported [52]. We counted the drought-related genes and a total of 115 were found in the DEGs, including 47 protein kinases, 30 plant endogenous hormones, 15 osmoprotectant synthases, 20 oxygen oxidoreductases and 3 acceptors. We summarized our drought-related DEGs and was shown in S10 Table. Huang also using transcriptomics to study drought responses in *Coix lacryma-jobi* Yiliao 5 [53]. In their study, 1,128 DEGs were identified that were less than our results identifying 5,315 DEGs. The results suggested that the *Coix* Yiliao 5 showed greater drought tolerance which respond to water stress with less gene expression. Meanwhile, they identified a total of 7,534 SSRs, which were also less than our results which identified 16,955 SSRs.

In our transcriptomic analysis, drought treatment increased transcript levels for 3,460 genes, and decreased those for 1,855 genes, as compared to the controls. It can be concluded that plants use a variety of mechanisms to regulate reproduction and maximize plant fitness and survival even under unfavourable conditions. Environmental stresses have a great impact on the yield of cereal crops. As detailed earlier, the effect of drought stress on yield is highly complex and involves processes as diverse as stem reserve accumulation, gametogenesis, fertilization, embryogenesis, and endosperm and grain development. Our present knowledge on these processes and on their mutual interactions is still scanty, especially if the potential impacts of environmental factors also have to be considered. Further application of modern research tools to reveal the complex molecular networks is urgently needed.

In conclusion, we initiated a transcriptome analysis of *Coix lacryma-jobi* based on the Illumina short-read sequencing platform in response to drought. In our study, 65,480 unique sequences and 5,315 DEGs were identified. We validated the genes via qRT-PCR and gene expression analysis. We also predicted SSRs from the transcriptome and detected SNPs within unigenes derived from the drought treatment and control samples, respectively. The candidate DEGs identified in this study could be applied to programs of molecular breeding that aim to produce plants with enhanced tolerance in the future. The data will also be very useful for the future study of gene regulation mechanism and molecular markers of drought resistance in *Coix*.

## Supporting information

S1 Table Sequence-specific primers used for qRT-PCR. (DOCX)

S2 Table The assembled unigenes and their functional annotation. (XLSX)

S3 Table List of unigenes of *Coix* annotated in COG for functional annotation and classification analysis. (XLSX)

S4 Table The list of GO function of all unigenes. (XLSX)

S5 Table The list of KEGG pathway annotation of all unigenes. (XLSX)

S6 Table The species distribution of the unigenes based on the NR database and the annotation of all the unigenes. (XLSX)

S7 Table The list of DEGs of *Coix* after drought treatment 7 days and the annotation. (XLSX)

S8 Table The list of GO function of the DEGs of *Coix* after drought treatment for 7 days. (XLSX)

S9 Table The list of KEGG pathway of *Coix* DEGs after drought treatment for 7 days. (XLSX)

S10 Table GO annotation number of DEGs related to drought in *Coix* transcriptome. (DOCX)

S11 Table MIQE checklist for authors, reviewers and editors. (XLS)
