# Supplementary material for: Transcriptome characterization and expression profile of Coix lacryma-jobi L. in response to drought
Source: PLoS One. 2021 Sep 3;16(9):e0256875. doi: 10.1371/journal.pone.0256875 (PMC8415600; doi:10.1371/journal.pone.0256875)
Supplement: S1 Table — (DOCX) [file pone.0256875.s001.docx]

**Table S1.** Sequence-specific primers used for qRT-PCR

| **Gene ID** | **Annotation** | **Primer-F (5'-3')** | **Primer-R (5'-3')** |
| --- | --- | --- | --- |
| CL6572.contig2 | *AP2/EREBP transcription factor* | CGGACGGAAGCTCCATTA | GGCAACGAGGACAACACTATC |
| CL7215.contig2 | *Protein phosphatase 2C* | TCCGCATGAGACTTGACC | AGCCACCAGAAACCAGAG |
| Unigene 10382 | *Dehydrin DHN1 protein* | CCTTGATTCCCTTCTTCCTCC | TCCAGCTCCAGCTCGTCTG |
| CL733.contig2 | *Protein SORBIDRAFT* | GATGCCACCACCAAAGG | CACTAGCGAAAGGAGAACG |
| CL6953.contig2 | *Serine/Threonine protein phosphatase* | GGCAAGCACCATTTCTACG | CATCCTGTCCTGGCACAT |
