# Supplementary material for: Transcriptome characterization and expression profile of Coix lacryma-jobi L. in response to drought
Source: PLoS One. 2021 Sep 3;16(9):e0256875. doi: 10.1371/journal.pone.0256875 (PMC8415600; doi:10.1371/journal.pone.0256875)
Supplement: S10 Table — (DOCX) [file pone.0256875.s010.docx]

| Enzymes or target | GO annotation number |
| --- | --- |
| Protein kinase (PKs) | 46 protein kinase activity (GO: 0004672) |
|  | 1 protein serine/threonine kinase activity (GO: 0000079) |
| Plant endogenous hormone (PEH) | 19 response to abscisic acid (GO: 0009737) |
|  | 4 response to cytokinin (GO: 0009735) |
|  | 7 response to ethylene (GO: 0009723) |
| Osmoprotectant synthases (OSs) | 14 sugar transmembrane transporter activity (GO: 0051119) |
|  | 92 sugar: hydrogen symporter activity (GO: 0005351) |
|  | 6 galactose transmembrane transporter activity (GO: 0005354) |
|  | 4 L-proline biosynthetic process (GO: 0055129) |
|  | 3 proline dehydrogenase activity (GO: 0004657) |
| Oxygen oxidoreductase (OOs) | 8 oxygen oxidoreductase activity (GO: 0052716) |
|  | 11 chitinase activity (GO: 0004568) |
|  | 3 methyltransferase activity (GO:0008168) |
|  | 1 NADPH binding (GO: 0070402) |
| Acceptor | 3 NAD or NADP as acceptor (GO: 0016616) |

**S10 Table DEGs related to drought in Coix transcriptome**
